# Zika Virus Neutralizing Antibody Responses Elicited by Vaccination or Infection

**DOI:** 10.1093/ofid/ofaf707

**Published:** 2025-12-03

**Authors:** Claudia M Galindo, Yun Ling, Eloi Kpamegan, Eduardo J M Nascimento, Erick Perez-Guzman, Juan P Aguilar Ticona, Kai Fern Chan, Priscila Castanha, Darunee Buddhari, Aaron Farmer, Stefan Fernandez, Whitney R Baldwin, Maima Kaiser, Jesuina Fernandes, Melissa Zahralban-Steele, Amanda Brinkman, Tim Rindfleisch, Kelley J Moss, Nadine Rouphael, Hana M El Sahly, Ernesto T A Marques, Albert I Ko, Camilo J Acosta

**Affiliations:** Vaccines Business Unit, Takeda Vaccines Inc., Cambridge, Massachusetts, USA; Vaccines Business Unit, Takeda Vaccines Inc., Cambridge, Massachusetts, USA; Vaccines Business Unit, Takeda Vaccines Inc., Cambridge, Massachusetts, USA; Vaccines Business Unit, Takeda Vaccines Inc., Cambridge, Massachusetts, USA; Vaccines Business Unit, Takeda Vaccines Inc., Cambridge, Massachusetts, USA; Fundação Oswaldo Cruz, Instituto Gonçalo Moniz, Salvador, Brazil; Vaccines Business Unit, Takeda Pharmaceuticals International, Singapore; Department of Infectious Diseases and Microbiology, University of Pittsburgh, Pittsburgh, Pennsylvania, USA; WRAIR-AFRIMS, Department of Virology, Bangkok, Thailand; WRAIR-AFRIMS, Department of Virology, Bangkok, Thailand; WRAIR-AFRIMS, Department of Virology, Bangkok, Thailand; Vaccines Business Unit, Takeda Vaccines Inc., Cambridge, Massachusetts, USA; Vaccines Business Unit, Takeda Vaccines Inc., Cambridge, Massachusetts, USA; Vaccines Business Unit, Takeda Vaccines Inc., Cambridge, Massachusetts, USA; Vaccines Business Unit, Takeda Vaccines Inc., Cambridge, Massachusetts, USA; Vaccines Business Unit, Takeda Vaccines Inc., Cambridge, Massachusetts, USA; Vaccines Business Unit, Takeda Vaccines Inc., Cambridge, Massachusetts, USA; Vaccines Business Unit, Takeda Vaccines Inc., Cambridge, Massachusetts, USA; Hope Clinic, Division of Infectious Diseases, Department of Medicine, Emory University School of Medicine, Atlanta, Georgia, USA; Departments of Molecular Virology and Microbiology and Medicine, Baylor College of Medicine, Houston, Texas, USA; Department of Infectious Diseases and Microbiology, University of Pittsburgh, Pittsburgh, Pennsylvania, USA; Fundação Oswaldo Cruz, Insituto Aggeu Magalhães, Recife, Brazil; Fundação Oswaldo Cruz, Instituto Gonçalo Moniz, Salvador, Brazil; Department of Epidemiology of Microbial Diseases, Yale School of Public Health, New Haven, Connecticut, USA; Vaccines Business Unit, Takeda Vaccines Inc., Cambridge, Massachusetts, USA

**Keywords:** correlate of protection, neutralizing antibody, TAK-426, vaccination, Zika

## Abstract

**Background:**

Zika virus (ZIKV) emergence in 2015–2016 was characterized by high attack rates and a wave of Congenital Zika Syndrome cases that affected several countries in the Americas. The sudden drop in virus transmission in the following years and the lack of a reliable correlate of protection have hampered the development of vaccines. ZIKV neutralizing antibodies (nAbs) responses to natural ZIKV infection provide insights into the potential efficacy of vaccine candidates.

**Methods:**

In this study, we compared anti-ZIKV nAb responses generated by a ZIKV vaccine (TAK-426), to those elicited by natural ZIKV infection in participants from diverse geographic areas using the same neutralizing antibody assay.

**Results:**

Those with a ZIKV infection (inapparent or symptomatic) exhibited higher levels of ZIKV nAbs, than TAK-426 vaccine recipients at all time points. The differences were less pronounced 1 month after TAK-426 dose 2. ZIKV nAb titers in vaccinated recipients were above the calculated threshold of protection at 1 month post-dose 2 for flavivirus (FV)-naive participants and at 1 and 6 months post-dose 2 for FV-primed participants. The kinetics of ZIKV nAbs were similar for both the natural infection and vaccination groups, exhibiting a peak, decline, and stabilization pattern; however, vaccine non-inferiority was not demonstrated.

**Conclusions:**

Our findings suggest that nAbs evoked by the current phase 1 formulation and dosage of TAK-426 may not protect against a ZIKV infection in endemic countries and that a booster dose should be further evaluated.

## Teaser

Delineating a Zika virus (ZIKV) correlate of protection is critical for vaccine development. Anti-ZIKV neutralizing antibodies (nAbs) elicited by natural infection were higher than those generated by TAK-426 vaccination, using the same assay. NAbs titers induced by vaccination, as well as natural infection, were above the calculated threshold of protection for 12 months. TAK-426 may not be protective in flavivirus endemic countries.

## Introduction

Half of the global population lives in regions that report Zika virus (ZIKV) cases [1–3]. ZIKV gained worldwide attention during the large outbreak in the Americas in 2015–2016 when it was associated with congenital birth defects that constitute Congenital Zika Syndrome [4]. Among these, microcephaly was the most severe, affecting 4% of infants exposed to ZIKV during gestation and 18.7% for functional neurological abnormalities with or without microcephaly [5]. Additionally, ZIKV infections can cause Guillain–Barré syndrome and other neurological sequelae [6]. Infection by ZIKV elicits neutralizing antibody (nAb) responses that mediate protection by targeting envelope proteins (especially domain III and quaternary epitopes) and blocking receptor-mediated virus entry and/or host membrane fusion; however, a reliable threshold of protection remains unknown [7].

Multiple vaccines in development, including Takeda's TAK-426, may serve as interventions in response to future ZIKV epidemics [8–10]. TAK-426 consists of an aluminum hydroxide adjuvanted purified, formalin-inactivated, whole ZIKV vaccine based on a plaque-purified sub-isolate of ZIKV strain PRVABC59 [11], which elicited robust nAbs that established complete protection in macaques [12]. TAK-426 was evaluated in a phase 1 clinical trial (ZIK-101) and demonstrated no clinically meaningful safety risks. At the highest vaccine dose of 10 µg, seropositivity for ZIKV nAbs was 100% at 1 year, and 94% and 76% at 2 years in flavivirus (FV)-naive and FV-primed participants, respectively [13]. The magnitude of nAb response elicited by the 10-μg TAK-426 dose predicted a probability of protection from infection of 90% among FV-naive phase 1 trial participants with a ZIKV nAb GMT ≥3.38 log10EC_50_ using a logistic regression with bias-reduction model and based on NHP data [14].

A traditional phase 3 vaccine efficacy study is challenging to perform because of low transmission rates of ZIKV, its mainly asymptomatic presentation, and the unpredictability of outbreaks. Therefore, an alternative clinical vaccine development approach, such as the US Food and Drug Administration's Accelerated Approval pathway for licensure, may be required, as was done previously for a chikungunya vaccine [15, 16]. For this approach, a ZIKV nAb titer would be used as a surrogate endpoint for protection against ZIKV infection. However, due to the absence of an immune marker of protection following ZIKV natural infection generated by long-term cohort studies, seroepidemiological studies can inform on this critical data gap.

In this study, we compared anti-ZIKV nAb responses generated by TAK-426 vaccination with those elicited by natural ZIKV infection. Natural infection samples were obtained from biorepositories of studies conducted in the Americas and Southeast Asia. These comprised participants with inapparent ZIKV infection and symptomatic participants (both non-severe and severe, including women who delivered infants with congenital malformations).

## MATERIALS AND METHODS

### Sources of Samples Collected From Individuals With Naturally Acquired ZIKV Infection (Table 1)

#### Brazil, Pau de Lima Cohort (ZIKV Inapparent Infections)

Biannual serosurveys from a cohort study in the dengue virus (DENV) endemic community of Pau de Lima (city of Salvador, situated in Northeast Brazil) have been performed since 2003 to investigate emerging infections [19–22]. In 2015, this area was the epicenter of a ZIKV outbreak with a high infection attack rate (>70%) [22]. While surveillance for arbovirus-like symptoms was not included, data on self-reported fever and rash were collected [22]. For this study, a subset of samples from adults (*n* = 54) who seroconverted during the outbreak defined by seropositivity for anti-ZIKV-non-structural protein 1 (NS1) IgG3 was selected, which included 5 serosurveys from 3 to 31 months post-ZIKV outbreak peak (Table 2).

**Table 1.** Participant Demographics and Past FV Exposure

	Pau De Lima Cohort, Brazil^a,b^	Travel Cohort, USA^a,c^		ZIK-101 Cohort, USA, And Puerto Rico ^d,e^		KFCS, Thailand^f^	Recife Cohort, Brazil^a,g^		Panel Puerto Rico, Ecuador, And The Caribbean
							ZIKV Symptomatic	Mothers with Newborns with Congenital Malformation	
FV or DENV Experience Before ZIKV Infection	FV-Primed	DENV-Exp.	DENV-Naive	FV-Primed	FV-Naive	Unknown	Unknown		Unknown
*N*	54	15	34	34	30	9	16	21	92
Female (%)	67	80	65	71	43	44	81	100	^h^
Age (years)	…	…	…	…	…	…	…	…	…
Median (min–max)	29 (18–50)	48 (25–66)	41 (18–68)	38 (21–49)	35 (20–49)	8 (2–76)	29 (21–57)	22 (15–38)	Adults
Mean (SD)	31 (8.9)	46 (13.9)	42 (14.9)	36 (8.5)	35 (8.4)	18 (23.8)	33 (10.8)	23 (6.1)	
Age group (%)	…	…	…	…	…	…	…	…	^h^
<30 y	56	7	29	26	27	7	50	91	
≥ 30 y	44	93	71	74	73	2	50	9	
Race (%)	…	…	…	…	…	^h^	^h^	^h^	^h^
Black	54	7	6	27	13				
White	7	67	79	71	83				
Brown	39	-	-	-	-				
Multiple	-	13	6	3	3				
Unknown	-	13	9	-	-				

^a^ZIKV nAb by RVP conducted at Takeda, Cambridge, MA, USA (2024).

^b^FV exposure prior to infection determined by FV Luminex (qualified) at Q2 Solutions, Durham, NC, USA (2024); all were FV-primed.

^c^DENV-experienced participants were defined as those with evidence of DENV-specific immunity on the first sample (enrollment) (DENV-specific nAb titers ≥250). DENV-naive participants were defined as those with no or only low-level cross-reactive DENV nAb titers (<250) [17].

^d^ZIKV nAb by RVP conducted at Takeda, Cambridge, MA, USA (2019–2021).

^e^FV exposure prior to first vaccine dose determined by FV Luminex (fit for purpose) at Q2 Solutions, Marietta, GA, USA (2019).

^f^Plasma samples tested with a ZIKV PRNT only; 8 of the 9 participants had previously received Japanese encephalitis vaccination.

^g^90% were DENV-experienced; based on the timing of sample collection and reactivity to DENV immunoglobulins G and M.

^h^Data not available.

DENV, dengue virus; FV, flavivirus; KFCS, Kamphaeng Phet Family Cohort Study; nAb, neutralizing antibody; RVP, reporter virus particle; SD, standard deviation; ZIKV, Zika virus.

**Table 2.** ZIKV nAbs in FV-primed Participants From Natural Infection in Pau de Lima and US Travel Cohort vs TAK-426 Vaccination (ZIK-101): GMTs and GMRs

ZIKV Infection		ZIKV Infection		Vaccine Recipients FV-Primed^c^		GMR (Pau De Lima/TAK-426)	GMR (US Travel Cohort)/TAK-426)
Pau de Lima FV-Primed^a^		US Travel Cohort DENV-Experienced^b^					
Months Post-ZIKV Outbreak	N	Days Since Symptom Onset	N	Time point	N	GMR (95% CI)	GMR (95% CI)
	GMT (95% CI)		GMT (95% CI)		GMT (95% CI)		
Prior	54	Prior	NA	Day of dose 1	33	1.3 (0.8–2.2)	NA
	460.0 (341.7–619.3)				357.8 (212.8–601.5)		
NA	NA	<30	5	1 m PD 1	34	NA	9.5 (1.9–47.4)
			23 847.5 (837.9–678 754.9)		2503.0 (1430.5–4379.8)		
3–7	54	30–90	8	1 m PD 2	34	2.8 (1.9–4.1)	5.5 (2.2–13.3)
	16 629.7 (13 359.2−20 701.0)		32 820.2 (9914.7–108 643.1)		6005.9 (4053.0–8899.9)		
10–12	54	90–180	14	6 m PD 2	34	4.1 (2.6–6.5)	5.7 (2.6–12.5)
	14 620.1 (11 635.7−18 369.8)		20 347.5 (11 549.7–35 846.7)		3587.0 (2198.1–5853.6)		
18–22	54	>180 and <360	11	12 m PD 2)	29	3.3 (2.1–5.3)	7.1 (3.2–15.8)
	5680.0 (4439.5– 7267.1)		12 323.2 (7368.8–20 608.6)		1726.1 (1058.5–2814.7)		
28–31	54	NA	NA	24 m PD 2	25	4.9 (3.3–7.4)	NA
	6767.1 (5550.6– 8250.1)				1373.9 (858.7–2198.4)		

^a^FV exposure prior to infection determined by FV Luminex (qualified) at Q2 Solutions, Durham, NC, USA (2024).

^b^DENV-experienced participants were defined as those with evidence of DENV-specific immunity on the first sample (DENV-specific nAb titers ≥250) [18].

^c^FV exposure prior to first vaccine dose determined by FV Luminex (fit for purpose) at Q2 Solutions, Marietta, GA, USA (2019).

CI, confidence interval; DENV, dengue virus; FV, flavivirus; GMT, geometric mean titer; GMR, GMT ratio; NA, not applicable; nAb, neutralizing antibody; PD, post dose; SD, standard deviation; ZIKV, Zika virus.

#### US Travel Cohort (ZIKV Symptomatic Infections)

Clinical data and specimens were collected from ZIKV-exposed participants aged 15–70 years living in the USA (*n* = 49) who developed symptomatic infection while traveling or in the limited transmission period in Florida [18]. ZIKV infection was established by ZIKV RNA identification in a body-fluid sample or ZIKV IgM and nAbs in serum with lower/no DENV nAbs. Participants had a maximum of 7 visits in 12 months, and past DENV infection was determined by the presence of DENV 1–4 nAbs.

#### Brazil, Recife Cohort (ZIKV Symptomatic Severe and Non-severe Infections*)*

##### Acute/Convalescent

ZIKV infection was confirmed in acute/convalescent (<30 days post onset of symptoms) samples from participants by reverse transcription polymerase chain reaction (RT-PCR) and/or IgM and/or plaque reduction neutralization assay (PRNT) [23, 24]. These febrile (≤72 hours) adults were recruited during the 2015–2016 ZIKV outbreak in northeast Brazil (Recife) at outpatient facilities (*n* = 16). Of these, 3 participants also had samples collected approximately 2 years after their illness.

##### Postpartum Period (Mothers of Infants With Microcephaly)

Samples were collected at delivery from ZIKV-infected mothers with neonates born with microcephaly at hospitals in Recife in 2015 [25]. The presence of prior ZIKV infection was confirmed by ZIKV IgM and/or PRNT (*n* = 21).

#### Thailand, Kamphaeng Phet Family Cohort Study (ZIKV Symptomatic Infections)

Samples from a longitudinal, febrile illness community dengue cohort study were obtained in 2015–2021 in the hyperendemic DENV area of Kamphaeng Phet province, north central Thailand [17,26]. Participants were DENV tested at enrollment, yearly, and for febrile illness (acute and convalescent). Samples identified as negative for DENV (either by serology or by polymerase chain reaction [PCR]) during the follow-up underwent ZIKV PRNT and ZIKV PCR testing, and 9 individuals were positive.

#### Panel (ZIKV Symptomatic Infections)

Commercially acquired serum samples (*n* = 92) from individuals residing in Puerto Rico, Ecuador, or the Caribbean were confirmed by ZIKV RNA in a body-fluid specimen or positive ZIKV IgM/nAb in serum [13]. Samples were drawn 7 days to 4 months after the onset of symptoms.

### Source of Samples Collected Following Immunization With TAK-426 (Table 1)

#### Preclinical [12, 27]

Serum samples from nonhuman primates (NHPs) from 2 studies were included (n = 26). Indian rhesus macaques received 2 doses of TAK-426 10 µg, intramuscularly, 28 days apart. Those dosed with commercially available FV vaccines (yellow fever, Japanese encephalitis, tick-borne encephalitis, and West Nile virus vaccines) before TAK-426 administration were considered the FV-primed (or -exposed) group.

#### Clinical [8, 13]

The phase 1 (ZIK-101) study included healthy FV-naive and FV-primed US adult participants, randomized to receive a 2-dose regimen of intramuscular TAK-426 of 2.5, 5, or 10 µg, administered 28 days apart. Blood samples were collected immediately prior to vaccination and at months 1, 2, 3, 7, 13, and 25 post-dose 1. Of the study participants, 64 received 2 doses of the 10-µg TAK-426 vaccine: 34 were FV-primed (mainly from Puerto Rico) and 30 were FV-naive participants, before vaccination.

### Immunoassays

The majority of the ZIKV nAb testing on the samples with naturally acquired ZIKV infection was done with a ZIKV reporter virus particle (RVP) microneutralization assay [28]. This assay was previously used for the TAK-426 NHP [23, 24] and phase 1 [8, 13] vaccine studies.

However, the ZIKV RVP assay could not be performed on the Kamphaeng Phet Family Cohort Study (KFCS) Thai samples because they were collected as plasma, which had not been established as a sample type for the RVP assay. Instead, we compared the existing ZIKV PRNT_50_ from Thailand with the ZIK-101 PRNT_50_ results [8, 13]. Methods and results are described in the Supplementary section.

When referencing the various cohort studies, we either use “primed” or “experienced” to highlight prior exposure to FV; both terms are used interchangeably throughout this article.

#### ZIKV RVP

Anti-ZIKV nAb levels in sera were measured using a fit-for-purpose ZIKV RVP microneutralization assay as described elsewhere [28]. The ZIKV RVP assay used positive control serum as part of the assay acceptance criteria and for tracking assay performance. Upper and lower control limits were established in 2019, and the same positive control sera and control limits were used throughout the duration of testing ZIK-101 samples (2019–2021) and natural infection samples (2024). Refer to the Supplementary section for further information.

#### FV Baseline Luminex

The assay was conducted to determine whether participants were FV-primed or FV-naive. It was performed on all samples before dosing with TAK-426 in the vaccine study and for the Pau de Lima cohort only, since samples were available prior to the ZIKV outbreak. The other groups (Recife, USA, and the panel) did not have samples collected before the outbreak.

It consists of a qualitative 11-plex immunoassay: ZIKV-NS1 (virus-like particle [VLP]), Yellow fever virus (YFV)-NS1, West Nile virus-envelope (purified protein), St. Louis Encephalitis virus (SLEV)-NS1, Japanese encephalitis virus-envelope, DENV1 and DENV4-envelope (VLP), and DENV2 and DENV3-NS1, and IgG control (Supplementary section)

### Statistical Analysis

Statistical analysis was performed using SAS® Version 9.4. Demographics for participants were summarized, and immune responses were analyzed using geometric mean titers (GMTs) after log_10_-transforming the ZIKV titers.

TAK-426 recipients and the Pau de Lima cohort were classified as either FV-primed or FV-naive based on the Luminex test, as samples were available prior to dosing or outbreak, respectively. The US cohort was classified as either DENV-experienced or DENV-naive prior to ZIKV infection, based on DENV nAb testing done during the acute or convalescent period. The Recife and panel groups were all assumed to be DENV-experienced.

Non-inferiority was demonstrated if the upper bound of the 95% confidence interval (CI) for the GMT ratio (GMR) (ZIKV-infected/TAK-426-vaccinated) at 1 month post-dose 2 among the FV-naive and the highest titer among ZIKV-infected participants, is less than the non-inferiority margin (NIM) of 2.0. Under prespecified assumptions, the statistical power for a comparison of 24 naturally ZIKV-infected participants and 24 TAK-426 vaccinees is 80%.

## RESULTS

### Demographics

In the nAb RVP analysis, 296 serum samples were included, of whom 232 belonged to the cohorts and commercial panel with a ZIKV infection, and 64 were TAK-426 vaccine recipients from the clinical trial. Demographics and past FV exposure are shown in Table 1. The median age was lowest in the Brazilian ZIKV-infected cohort (22–29 years), compared with the vaccines (35–38 years) and the US ZIKV-infected cohort (41–48 years). Most individuals were female, and the Brazilian Pau de Lima cohort identified as predominantly Black or Brown, whereas the US group and TAK-426 vaccine recipients predominantly identified as White. There were no FV-naive participants in the Pau de Lima cohort based on the FV Baseline Luminex results, tested on samples obtained prior to the ZIKV outbreak. Indeed, serological data from this geographic area indicate that by age 10 years, 80% of the population are DENV seropositive [22]; we assumed that all Recife samples would also be DENV positive. The panel of adults included only the country of origin (Puerto Rico, Ecuador, and the Caribbean).

### ZIKV nAb GMTs: ZIKV-Infected vs TAK-426

**Figure 1.**
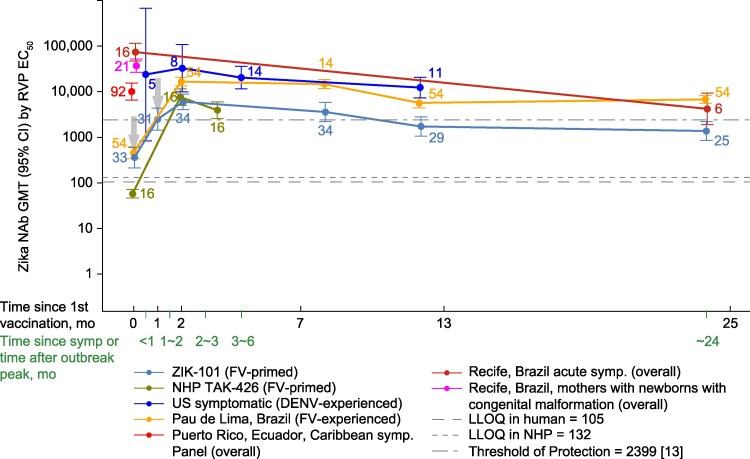
ZIKV neutralizing antibody GMTs by RVP in natural infection and vaccination in FV-primed cases (all ages). The data point numbers refer to the sample size. CI, confidence interval; DENV, dengue virus; EC_50,_ effective concentration 50%; FV, flavivirus; GMT, geometric mean titer; LLOQ, lower limit of quantification; mo, month; nAb, neutralizing antibody; NHP, nonhuman primate; RVP, reporter virus particle; symp, symptom; and ZIKV, Zika virus.

**Figure 2.**
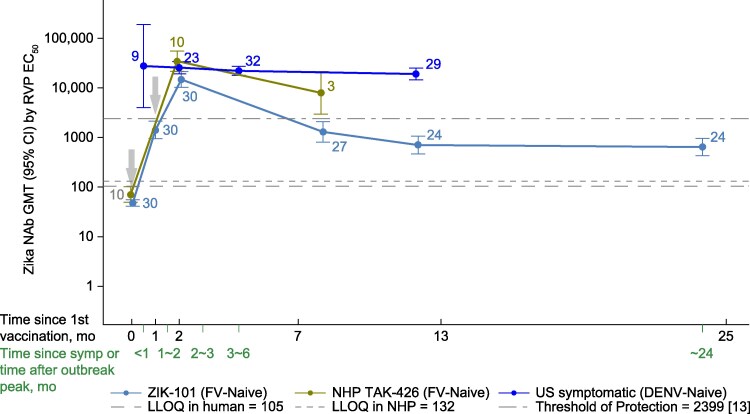
ZIKV neutralizing antibody GMTs by RVP in natural infection and vaccination in DENV-naive or FV-naive participants (all ages). The data point numbers refer to the sample size. CI, confidence interval; DENV, dengue virus; EC_50_, effective concentration 50%; FV, flavivirus; GMT, geometric mean titer; LLOQ, lower limit of quantification; mo, month; nAb, neutralizing antibody; NHP, nonhuman primate; RVP, reporter virus particle; symp, symptom; and ZIKV, Zika virus.

Considerably higher ZIKV nAb titers were observed at all time points in all ZIKV-infected groups at comparable time points among the TAK-426 recipients (both humans and NHPs) (Figures 1 and 2, and Table 2). This effect was more pronounced among FV-experienced (Figure 1) compared with FV-naive (Figure 2) individuals.

Among the FV or DENV primed, the highest antibody levels were detected in the acute/convalescent samples of the symptomatic participants from Recife, with GMTs of 74 308.2 (95% CI: 44 043–125 370) and 38 371 (95% CI: 2661–55 331) in women at delivery (Figure 1). Symptomatic US participants (30–90 days post-symptom onset) had GMTs of 32 820.2 (95% CI: 99 147–108 643.1) (Table 2). The ZIKV-infected (Pau de Lima) group displayed GMTs of 16 629.7 (95% CI: 13 359.2–20 701.0), observed 3 to 7 months after the infection (Table 2). The ZIKV-infected panel with a single time point exhibited the lowest values among the infected (10 052.0 [95% CI: 6572.4–15 373.7]) (Figure 1). The titers elicited by the vaccine were the lowest of all those tested, with a peak of GMTs of 6005.9 (95% CI: 4053.0–8899.9), 2 months post-dose 2 among the FV-primed (Table 1). Among the FV or DENV-naive, the US participants had GMTs of 25 674.9 (95% CI: 19 316.7–34 125.8) while TAK-426 induced lower GMTs of 14 790.5 (95% CI: 10 288.9–21 261.6). ZIKV nAb titers in vaccinated recipients were above the calculated threshold of protection at 1 month post-dose 2 for FV-naive participants and at 1 and 6 months post-dose 2 for FV-primed participants.

Over time, a decline in nAb titers was observed among the ZIKV-infected cohorts, followed by stabilization. Likewise, 1 month after the TAK-426 second dose, nAb titers decreased and stabilized after 12 months; ZIKV nAbs titers among the naive vaccine recipients were always lower than the FV-primed recipients except at 1 month post dose 2. The nAb titers by age groups (<30 and ≥30 years) can be found in Supplementary Figures 2*A*, 2*B*, 3*A*, and 3*B*, with no significant differences when compared with the overall data.

### ZIKV nAbs GMRs: ZIKV Infected vs TAK-426

#### FV-Naive

The GMR (DENV-naive US travel cohort/FV-naive ZIK-101) was 1.7 (95% CI: 1.0–3.0 [ Table 3]) at a comparable time point of 1 month post-dose 2, where vaccine titers reached their highest level. Non-inferiority was not established for TAK-426 as the upper bound of the 95% CI of the GMR was not less than the NIM of 2.0. Non-inferiority analysis among the Pau de Lima population was only conducted on the FV-primed participants (see below) due to the unavailability of FV-naive participants.

**Table 3.** ZIKV nAbs in FV-naive Participants From Natural Infection in US Travel Cohort vs TAK-426 Vaccination (ZIK-101): GMTs and GMRs

ZIKV Infection		Vaccine Recipients FV-Naive^b^		GMR (US Travel Cohort)/TAK-426)
US Travel Cohort DENV-Naive^a^				
Days Since Symptom Onset	N	Time point	N	GMR (95% CI)
	GMT (95% CI)		GMT (95% CI)	
Prior	NA	Day of dose 1	30	NA
			47.7 (40.9–55.7)	
<30	9	1 m PD 1	30	19.4 (6.5–58.5)
	27 584.9 (4008.2–189 842.4)		1419.3 (942.4–2137.5)	
30–90	23	1 m PD 2	30	1.7 (1.1–2.7)
	25 674.9 (19 316.7–34 125.8)		14 790.5 (10 288.9–21 261.6)	
90–180	32	6 m PD 2	27	17.0 (10.7–27.0)
	22 089.6 (17 909.4–27 245.5)		1297.7 (808.9–2081.9)	
>180 and <360	29	12 m PD 2)	24	27.1 (17.3–42.4)
	19 081.2 (14 550.1–25 023.4)		704.6 (465.2–1067.3)	
NA	NA	24 m PD 2	24	NA
			642.1 (431.8–954.7)	

^a^DENV-naive participants were defined as those with no or only low-level cross-reactive DENV nAb titers (<250) [18].

^b^FV exposure prior to first vaccine dose determined by FV Luminex (fit for purpose) at Q2 Solutions, Marietta, GA, USA (2019).

CI, confidence interval; DENV, dengue virus; FV, flavivirus; GMT, geometric mean titer; GMR, GMT ratio; NA, not applicable; nAb, neutralizing antibody; PD, post dose; SD, standard deviation; ZIKV, Zika virus.

#### FV-Primed

Although non-inferiority analysis is usually not performed among populations with past infections, we report that the GMR (DENV-experienced US travel cohort/FV-primed in ZIK-101) was 5.5 (95% CI: 2.5–11.7 [Table 2]) at a comparable time point of 1 month post-dose 2, where vaccine titers reached their highest level. Non-inferiority was also not established for TAK-426.

The GMR (FV-primed participants in Pau de Lima and ZIK-101) was 2.8 (95% CI: 1.9–4.1 [Table 2]) at a comparable time point of 1 month post-dose 2, where vaccine titers reached their highest level. Non-inferiority was not established for TAK-426 as the upper bound of the 95% CI of the GMR was not less than 2.0. A multivariable analysis was conducted to obtain GMRs adjusted for sex and age for both the vaccinated group and the Pau da Lima cohort; however, even after adjustment, the non-inferiority outcome was not achieved.

## DISCUSSION

To our knowledge, this is the first time that a vaccine-induced versus natural infection comparison of nAb responses, measured over time with the same assay, has been reported for ZIKV. The magnitude and kinetics of the ZIKV nAbs elicited by either 2 doses of 10 µg of TAK-426 vaccination or a natural ZIKV infection were assessed using the same assay (mainly by RVP). Participants with or without evidence of FV exposure before ZIKV infection exhibited consistently higher levels of ZIKV nAbs than the TAK-426 vaccine recipients at all time points. The differences were less pronounced 1 month after dose 2 of TAK-426, although the non-inferiority of TAK-426 was not demonstrated. The kinetics of ZIKV nAbs were similar in both natural infection and vaccination groups, showing peak, decline, and stabilization patterns that usually ranged 1–2, 2–12 months, and 12–24 months, respectively, since onset of symptoms or time since outbreak peak or first vaccination. Similar findings of a decline in ZIKV nAb response approximately 2 years after ZIKV infection have been reported [29]. ZIKV nAb titers were above the calculated threshold of protection [9] at all time points and for vaccine recipients at 1 month (among FV-naive participants) and at 1 and 6 months (among FV-primed participants) post-dose 2.

NAbs have been identified as correlates of protection for vaccines targeting other FVs, including Japanese encephalitis, yellow fever, and tick-borne encephalitis viruses [30]. This seems to be applicable for ZIKV, since an efficacy study with TAK-426 determined an nAb correlate of protection in macaques [12] and extrapolated it to humans [9]. Delineating a ZIKV nAb titer that correlates with protection in humans against ZIKV remains a critical gap in vaccine development. ZIKV nAb titers in the current study were above this threshold of protection (3,38 log_10_, equivalent to a titer of 2399) at all time points for the ZIKV-infected cohorts and for vaccine recipients at 1 through 6 months (FV-primed) and 1 month (FV-naive), post-dose 2.

Selection of a Zika vaccine candidate should not rely solely on high antibody responses but should also consider the potential for vaccine-associated antibody-dependent enhanced disease [31]. This phenomenon is a key concern in vaccine development for both Zika and dengue, as a prior ZIKV infection may heighten the risk of severe dengue [31, 32], and preexisting DENV immunity may lead to ZIKV severe outcomes [33].

Though the same neutralization assay was used for most samples, there are distinctions among the ZIKV-infected human cohorts. The symptomatic cohorts tested positive for either RT-PCR or E protein-based IgM/IgG serology, while the “inapparent infection” cohort tested positive on the NS1 assay. Nevertheless, there is a high correlation between the presence of NS1 and neutralizing antibodies [34], indicating that NS1 seropositivity reflects prior ZIKV exposure. Additionally, those samples characterized as FV-primed samples were based on a mixture of orthoflavivirus antigens (VLP, E protein or NS1), but seroprevalence data reflect that well above 50% of the population living in DENV-endemic areas have evidence of DENV exposure by adolescence or adulthood [22, 35]. The NHP data are presented to provide context for human data but the two are not combined.

When comparing a new candidate vaccine with a vaccine that demonstrated efficacy (unlike TAK-426), regulatory authorities generally recommend a GMT ratio of 1.5 or 2.0. However, since the ZIKV nAb titers were based on serial dilutions, variations of 1 dilution more or 1 dilution less fall within the margin of error for dilution assays. A difference of less than 4-fold may not be meaningful, and the immune responses observed in the TAK-426 vaccine recipients, at the time of peak titers, were nearly equivalent to those in the ZIKV-infected cohorts.

Another limitation to our findings is that ZIK-101 and panel serum samples were tested by RVP in 2019 through 2021, whereas the remaining cohorts were tested in 2024. Nonetheless, we do not believe this adversely impacted the results because identical lots of critical reagents and assay controls were maintained from 2019 to 2021. Furthermore, the tracking and trending data of controls suggested the data output was consistent over time (Supplementary Figure 4).

The titers observed in the panel were comparable to the titers elicited by the TAK-426 vaccine recipients. Similarly, a phase 1 study involving an mRNA-based ZIKV vaccine produced nAb titers that were comparable to those found in convalescent sera [30]. However, the samples collected from convalescent individuals at a single time point lack thorough information regarding the time of collection and the history of past exposures to FV. In contrast, the cohorts used in our study were well characterized.

This study, using available serum banks from diverse geographic areas, demonstrated that nAb titer responses induced by ZIKV infection are higher than those generated by TAK-426 vaccination. These TAK-426 titers remained above the protective threshold for up to 6 months after dosing the FV-primed recipients. Our findings suggest that the current formulation and dose of TAK-426 might not protect against ZIKV infection in endemic regions beyond 6 months and for at least 1 month among FV-naive individuals. Dosing individuals previously vaccinated with a licensed dengue vaccine, a higher dose (>10 µg), or a booster dose may yield ZIKV nAb levels comparable with those observed in ZIKV-infected individuals. TAK-426 cellular immune responses such as CD8 + cells were not measured and may play a role in protection alongside the humoral responses. Future studies have been discontinued, given that the Biomedical Advanced Research and Development Authority (BARDA) funds that supported ZIKV vaccine research and development have expired.

## Supplementary Material

ofaf707_Supplementary_Data

## Supplementary Data

Supplementary materials are available at *Open Forum Infectious Diseases* online. Consisting of data provided by the authors to benefit the reader, the posted materials are not copyedited and are the sole responsibility of the authors, so questions or comments should be addressed to the corresponding author.
